# Fabrication of a Nano-ZnO/Polyethylene/Wood-Fiber Composite with Enhanced Microwave Absorption and Photocatalytic Activity via a Facile Hot-Press Method

**DOI:** 10.3390/ma10111267

**Published:** 2017-11-03

**Authors:** Baokang Dang, Yipeng Chen, Xiaoping Shen, Bo Chen, Qingfeng Sun, Chunde Jin

**Affiliations:** 1 School of Engineering, Zhejiang A&F University, Hangzhou 311300, China; dang972028790@163.com (B.D.); 18868195633@sina.cn (Y.C.); sxp1031@hotmail.com (X.S.); 2 Zhejiang New Wood Material Technology Co., Ltd., Ningbo 315300, China; cb19871001@126.com; 3 Key Laboratory of Wood Science and Technology, Hangzhou 311300, China

**Keywords:** wood fiber composite, photocatalytic activity, microwave absorption, thermomechanical

## Abstract

A polyethylene/wood-fiber composite loaded with nano-ZnO was prepared by a facile hot-press method and was used for the photocatalytic degradation of organic compounds as well as for microwave absorption. ZnO nanoparticles with an average size of 29 nm and polyethylene (PE) powders were dispersed on the wood fibers’ surface through a viscous cationic polyacrylamide (CPAM) solution. The reflection loss (RL) value of the resulting composite was −21 dB, with a thickness of 3.5 mm in the frequency of 17.17 GHz. The PE/ZnO/wood-fiber (PZW) composite exhibited superior photocatalytic activity (84% methyl orange degradation within 300 min) under UV light irradiation. ZnO nanoparticels (NPs) increased the storage modulus of the PZW composite, and the damping factor was transferred to the higher temperature region. The PZW composite exhibited the maximum flexural strength of 58 MPa and a modulus of elasticity (MOE) of 9625 MPa. Meanwhile, it also displayed dimensional stability (thickness swelling value of 9%).

## 1. Introduction

Wood polymer composites (WPCs) are used for building materials, furniture, packaging, etc., because of its dimensional stability, high strength, hydrophobic properties, and corrosion resistance [1,2,3]. Generally, the components of a WPC are preblended in a mixer, extruded by a single twin-screw extruder, and finally molded by an injection molder [4]. The components of a WPC should be dried at a certain temperature to reach a constant weight, before processing. However, the increased cost of plastic and the limited availability of wood fibers have restricted WPCs’ development [5]. Wood-fiber composites filled with plastic could be obtained using a mechanical grind and a hot-press. In order to solve the incompatibility between the fibers and the polymers, amide compounds could be used to form linking bridges [6].

In order to develop the potential commercial application of WPCs, nanomodification and interfacial coupling could be implemented [7,8,9,10]. Inorganic nanoparticles used in WPCs could enhance the functionalized commercial applications of WPCs. The special properties of ZnO nanoparticles (NPs) were investigated, including catalysis, optics, magnetism, mechanics, etc. [1,11,12,13]. It was concluded that ZnO NPs could be used for UV shielding materials, antibacterial agents, fluorescent materials, photocatalytic materials, and microwave absorbing materials [14,15,16,17,18]. Patterson et al. prepared aramid fiber composites through ZnO NPs coating; this treatment increased the interfacial strength and improved the resistance to UV irradiation [13]. Wang et al. fabricated the reduced graphene oxide/ZnO composites through self-assembly and in-situ photo-reduction. The product exhibited photocatalytic activity in degrading Rhodamine B (RhB) [15]. Wang et al. synthesized flower-like, ZnO-coated Ni nanoparticles via an atomic layer deposition method. The optimal reflection loss was −48 dB at 10.4 GHz [19].

In this study, we describe the fabrication of a PE/ZnO/wood-fiber composite through a grinding process and a hot-press. ZnO NPs were deposited on the fibers’ surface during the grinding process. The results indicated that the ZnO NPs not only enhanced the mechanical strength of the product, but also provided it with multifunctional applications. The PE/ZnO/wood-fiber composite showed microwave absorption properties and UV light photocatalytic activity. The effects of ZnO on the storage modulus and damping properties of the PE/ZnO/wood-fiber composite were investigated.

## 2. Results and Discussion

**Figure 1:**
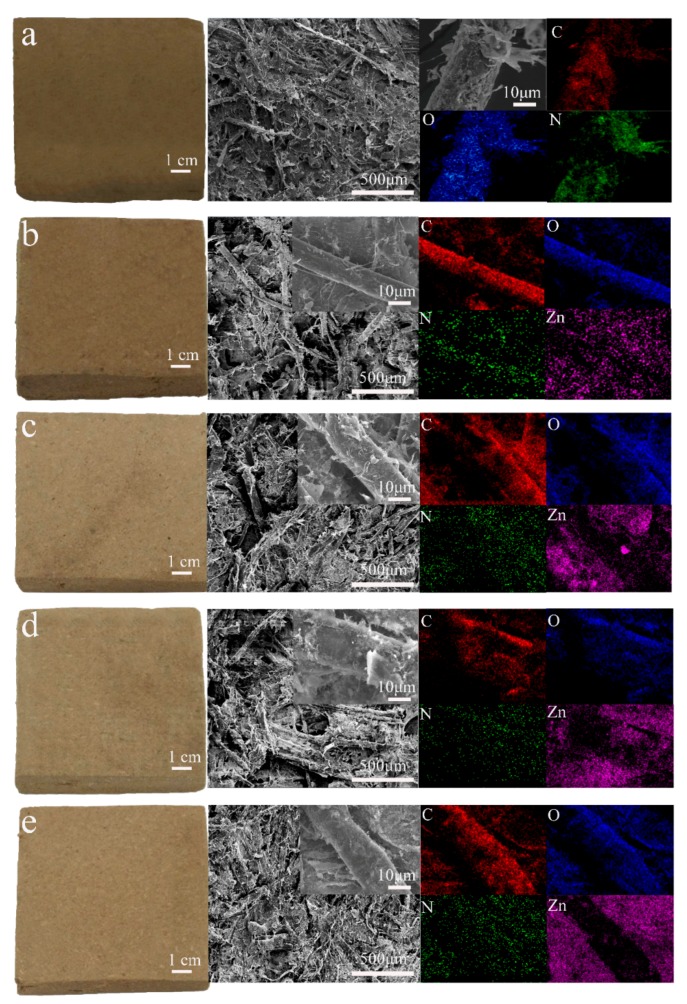
A digital image, SEM, and EDX mapping of the PE/wood-fiber composite and of the PE/ZnO/wood-fiber composite (PZW). (**a**) PZW0; (**b**) PZW1; (**c**) PZW2; (**d**) PZW3; (**e**) PZW4.

Figure 1 shows the macromorphology, SEM, and energy-dispersive X-ray spectroscopy (EDS) elemental mapping of all composite samples. The macromorphology, SEM image, and EDS mapping of the PZW0 composite (PE/wood fiber composite without ZnO) is shown in Figure 1a. The color of PZW0 was similar to that of the fibers. In the middle SEM image of Figure 1a, the fibers were stacked together, and the PE powder was attached to the fibers. At 5000× magnification, the PE and microfiber sheets were visible on the fibers’ surface. EDS mapping detected the main element composition of PZW0; the main elements were C, O, and N. Figure 1b displays the appearance and micromorphology of PZW1 (PE/ZnO/wood fiber composite with 2% ZnO). The color of PZW1 faded slightly compared to that of PZW0. This is because the ZnO NPs that were dispersed in the composite became milky-white, causing PZW1 to fade slightly. In the inset of the SEM image, PE and few ZnO NPs are visible on the surface of the fibers. The ZnO NPs were deposited on the fibers by hydrogen bonding, which formed between the hydroxyl groups of cellulose and the water bound on the surface of the ZnO NPs. The PE powders were dispersed onto the fibers’ surface by flocculation and physical adsorption of the cationic polyacrylamide (CPAM) suspension. The main elements of PZW1 were measured by EDS mapping and resulted to be C, N, O, and Zn. Zn was uniformly distributed on the fibers, which was beneficial to increase their mechanical strength. Figure 1c–e display the morphology and EDS mapping of PZW2 (PE/ZnO/wood fiber composite with 4% ZnO), PZW3 (PE/ZnO/wood fiber composite with 6% ZnO), and PZW4 (PE/ZnO/wood fiber composite with 8% ZnO). As the concentration of ZnO increased, the nanoparticles on the surface of the PZW composites increased.

**Figure 2:**
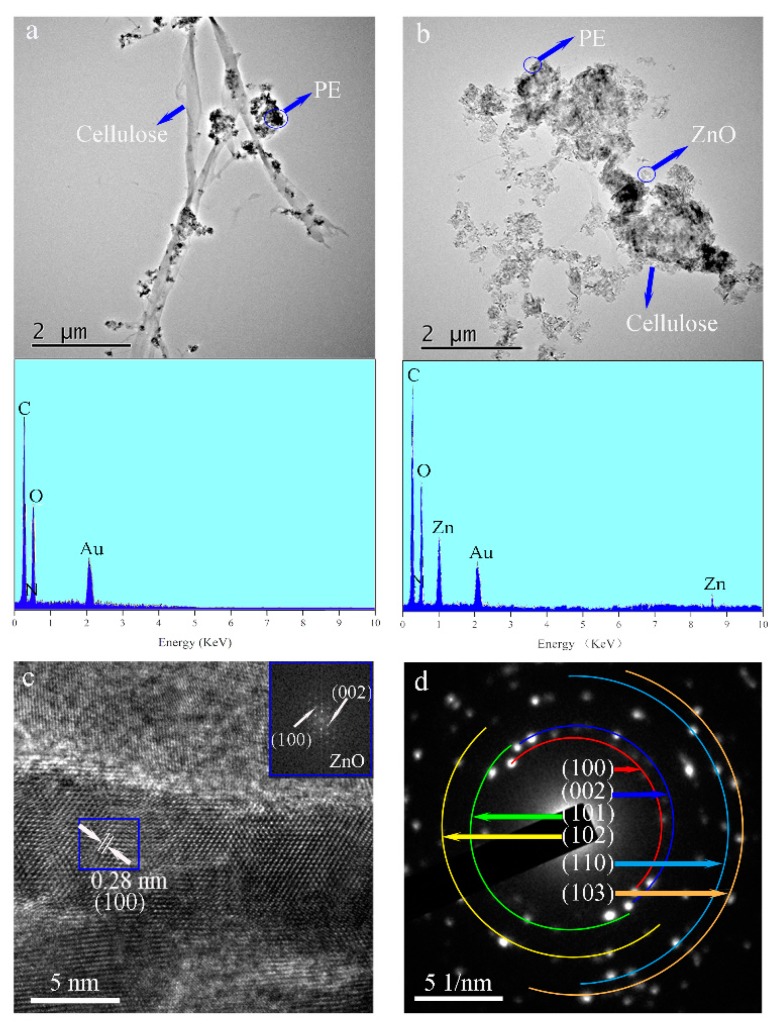
TEM images and EDS mapping of the PE/wood fiber-composite (**a**), and of the PE/ZnO/wood-fiber composite (**b**); (**c**) HRTEM image of the PZW composite, and SAED image corresponding to the area in the blue rectangle (inset); (**d**) SAED pattern of the PZW composite.

Figure 2 shows TEM images of the PE/wood-fiber composite and of the PE/ZnO/wood-fiber composite. As shown in Figure 2a, the microfibers were connected to the fibers; the average diameter was 300 nm. Meanwhile, PE particles were distributed on the microfibers. As detected by EDS analysis shown in the inset, the main elements were C, derived from PE and wood fiber, with atomic percentage of 62%, and N, provided by the amino group of CPAM, with atomic percentage of 3%. ZnO NPs were distributed around the fibers, as seen at low resolution in Figure 2b, and the presence of Zn was detected by EDS analysis, as shown in the inset. The average crystalline size of the ZnO NPs was 29 nm, which was calculated from the TEM images. The elements C, N, O, and Zn were detected in the EDS spectrum, with corresponding atomic percentages of 50%, 2%, 27%, and 21%, respectively. The presence of the ZnO NPs confirmed that ZnO NPs were loaded on the fibers by mechanical and chemical action. A high-resolution TEM image in Figure 2c shows lattice fringes. The crystal lattice had a d-spacing (the spacing between planes in crystal) of 0.28 nm, which corresponded to the interspace of the (100) plane. The element diffraction pattern was obtained by fast Fourier transform of the blue line area of the high resolution TEM (HRTEM) image, as shown in the inset of Figure 2c. The (100) and (002) lattices of ZnO are indicated in the inset. The observed diamond patterns indicated that ZnO was polycrystalline in nature [20]. Figure 2d shows the electron diffraction (SAED) pattern of the PE/ZnO/wood-fiber composite. The bright diffraction spots confirmed the high crystallinity of ZnO, corresponding to wurtzite structure.

**Figure 3:**
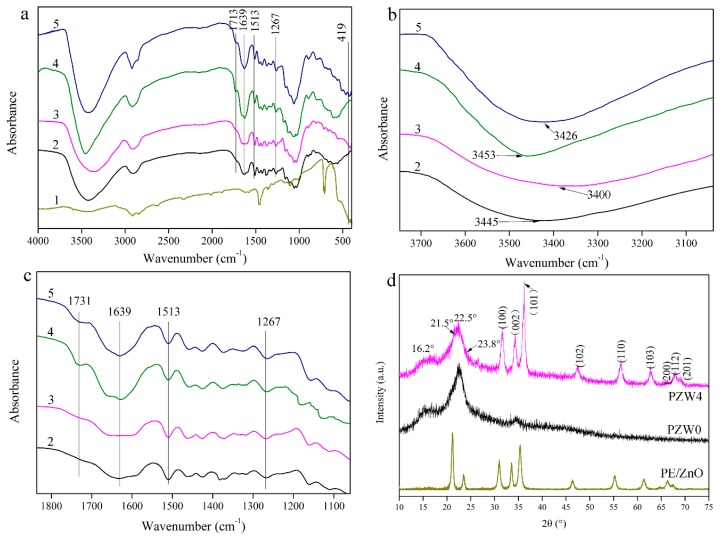
(**a**–**c**) The FTIR spectra of PE/ZnO (1), PZW0 (2), PZW4 (3), PZW0 without hot-pressing (4), and PZW4 without hot-pressing (5); (**d**) The XRD patterns of the PE/ZnO, PZW0, and PZW4 composites.

Figure 3a shows the functional group structure of the PE/ZnO composite and of the PZW composite. In Figure 3a, the absorption band at 3400 cm^−1^ was ascribed to the OH stretching vibration [21]. The peak at 1731 cm^−1^ was attributed to the C=O stretching vibration, which was a characteristic peak of hemicellulose [22,23]. The absorption peak at 1639 cm^−1^ was assigned to the bending vibration of water absorption and to the C=O stretching vibration of the amide group [24]. The stretching vibration of the aromatic ring skeleton at 1513 cm^−1^ corresponded to the characteristic peak of lignin [25]. The absorption band at 1267 cm^−1^ was ascribed to the C–O stretching vibration of the ester and to the C–N stretching vibration [26]. The peak at 419 cm^−1^ was attributed to the metal-oxide bond [12]. Figure 3b shows the changes of the hydroxyl groups in the range of 3200–3600 cm^−1^ of the FTIR spectrum. Compared to the FTIR spectra of the PZW0 composite without hot-pressing, the absorption peak of the hydroxyl group of the PZW4 composite without hot-pressing tended to widen and shift to lower wavenumbers. It was observed that the hydroxyl groups of the PZW4 composite without hot-pressing formed hydrogen bonds after loading the ZnO nanoparticles. After hot-pressing, the absorption peak of the hydroxyl group of the PZW composite widened, compared with the corresponding peak of the PZW composite without hot-pressing. It was observed that the hydroxyl groups bonded during hot-pressing. Meanwhile, the absorption peak of the PZW4 composite shifted to the low wavenumbers (3400 cm^−1^) as the ZnO NPs were loaded. This indicated that the hydroxyl groups on the ZnO NPs surface were linked via hydrogen bonding. Figure 3c shows the changes of the different chemical groups in the range of 1100–1800 cm^−1^ of the FTIR spectra. Compared with the PZW composite without hot-pressing, the peak at 1731 cm^−1^ weakend and dispersed in the FTIR spectra of the PZW composite. It was described that part of the xylose released from hemicellulose could be degraded at high temperature. The reaction of the aromatic ring of lignin with the degradation products of xylose form an adhesive. The peak at 1267 cm^−1^ of the PZW composite widened, compared with that of the PZW composite without hot-pressing. The peak of the ester demonstrated that the adhesive was produced after hot-pressing. The ZnO NPs could also be attached to the composite via the adhesive.

Figure 3d displays the crystalline structure of PE/ZnO, PZW0, and PZW4. The diffraction peaks at 16.2° and 22.5° were related to the (101) plane and the (002) plane of cellulose [1,27]. The XRD peaks at 21.5° and 23.8° corresponded to the (100) and (200) planes of PE [28]. These peaks indicated that PE was attached to cellulose. For the PZW0 composite, there were only characteristic peaks of cellulose and PE on the XRD curve. The other diffraction peaks that appeared at 31.6°, 34.3°, 36.2°, 47.4°, 56.6°, 62.7°, 66.4°, 67.9°, and 69.3° were matched to the (100), (002), (101), (102), (110), (103), (200), (112), and (201) planes of the wurtzite structure (PDF No. 36-1451), respectively. [1] It was confirmed that the ZnO NPs were attached to cellulose. The lattice parameters of the hexagonal wurtzite phase were described as a = b = 0.325 nm, c = 0.521 nm, α = β = 90°, and γ = 120°. The crystalline structure of cellulose did not change as ZnO was added. The XRD patterns of the PE/ZnO composite showed the diffraction peaks of PE and ZnO. This indicated that the ZnO NPs were loaded onto the PE/ZnO composite via an extrusion procedure.

**Figure 4:**
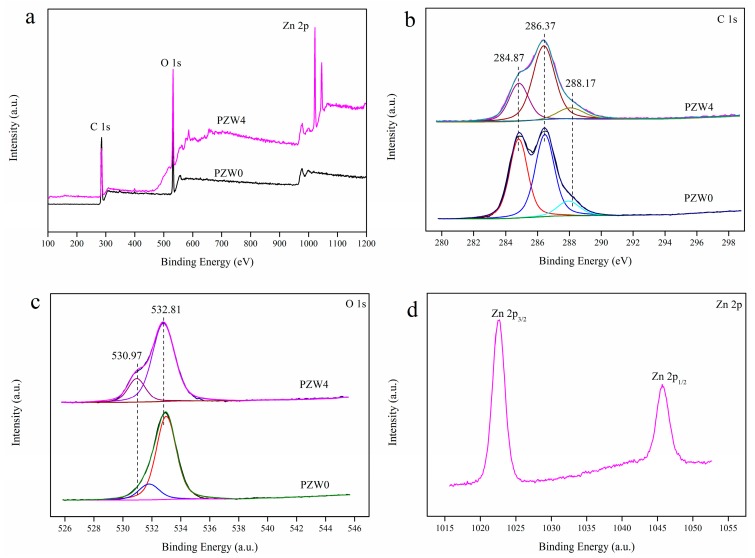
Survey XPS spectra of PZW0 and PZW4 (**a**); C 1s (**b**); O 1s (**c**); and Zn 2p (**d**).

Figure 4 shows the X-ray photoelectron spectroscopy (XPS) spectra of the PZW0 and PZW4 composites; the atomic content and the C/O ratio, showing the elimination of carbon as a contaminant, are listed in Table 1. The binding energies obtained in the XPS analyses were corrected for specimen charging by referencing C 1s to 284.6 eV. After eliminating the contaminant carbon, the atomic percentage of Zn was 2.49%, 4.01%, 6.35%, and 8.81%, respectively. The survey XPS spectra of PZW0 displayed two binding-energy bands of C 1s and O 1s, as shown in Figure 4a, while the XPS spectra of PZW4 showed four binding-energy bands of C 1s, O 1s, Zn 2p_3/2_, and Zn 2p_1/2_. These results further confirmed that ZnO was coated on the fibers. In Figure 4a, the binding energy band at 401.17 eV was attributed to N 1s, which was derived from amide group of CPAM. The atomic content and C/O ratio were determined by XPS analysis, and the resulting data are listed in Table 1. As the concentration of the ZnO NPs increased, the relative atomic content of O increased and the C/O ratio decreased. Figure 4b displays the C 1s spectra of PZW0 and PZW4. The binding energies of 284.87, 286.37, and 288.17 eV were ascribed to the C–C bond, the C–O bond, and the C=O bond, respectively [15]. Compared with the relative peak intensity of the C–C bond in PZW0 in Figure 4b, the relative intensity of the C–C bond in PZW4 decreased. The O 1s spectrum is displayed in Figure 4c; the peak with the binding energy of 532.81 eV was attributed to the O–H bond and to water absorption [29]. An additional peak with binding energy of 530.97 eV appeared, which was ascribed to the Zn–O bond [12]. The binding energies of 1022.57 and 1045.67 eV were assigned to Zn 2p_3/2_ and Zn 2p_1/2_, respectively, and the difference of the binding energies, corresponding to 23.1 eV, matched the standard value for ZnO [12].

**Figure 5:**
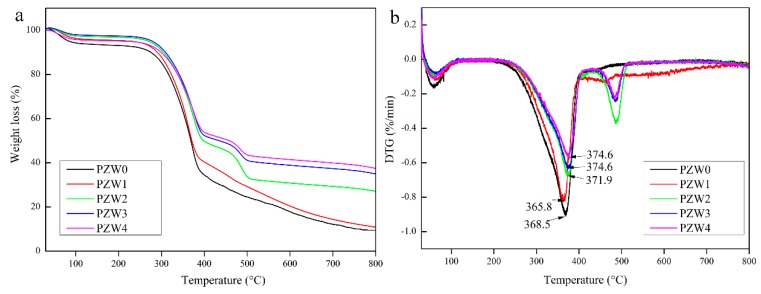
The TG (**a**) and DTG (**b**) curves of PZW0, PZW1, PZW2, PZW3 and PZW4.

Figure 5 shows the thermogravimetric (TG) and differential thermogravimetric (DTG) curves of PZW0, PZW1, PZW2, PZW3, and PZW4. All the TG curves could be divided into three stages, as shown in Figure 5a. A weight loss of 5% occurred below 100 °C and was attributed to the evaporation of residual moisture. The second stage was due to the thermal decomposition of wood fibers, which occurred at temperatures in the range of 250–420 °C. The third stage was the decomposition process of PE and occurred at temperatures in the range of 450–550 °C [30]. The main reason of the third stage could be ascribed to the fact that the ZnO NPs could transfer the heat and disturb the spread of degradation components. DTG decomposition curves of all PZW composites are shown in Figure 5b. The DTG curve of PZW0 shows a rapid decomposition of wood fibers occurring at a temperature of 368.5 °C. As the concentration of ZnO increased, the decomposition temperature of wood fibers was 365.8 °C, 371.9 °C, 374.6 °C, and 374.6 °C, respectively. These results showed that ZnO had a flame-retardant effect on the wood-fiber composites.

**Figure 6:**
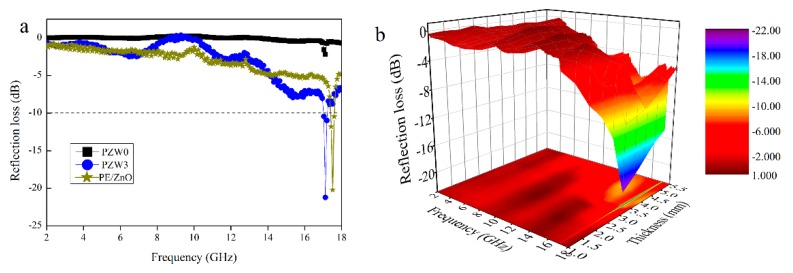
The reflection loss curves (**a**) of PZW0 and PZW3 with a thickness of 3.5 mm in the frequency range of 2–18 GHz; The three-dimensional representation (**b**) of the reflection loss of PZW3.

Figure 6a shows the comparison of the reflection loss curves of the PE/ZnO composite, the PZW0 composite, and the PZW3 composite, with a thickness of 3.5 mm in the frequency range of 2–18 GHz. The minimum reflection loss (RL) value of PZW0 was −2 dB at 17.17 GHz, while the minimum RL value of PZW3 was −21.21 dB in the same frequency. The minimum RL value of the PE/ZnO composite was 19 dB in the frequency of 2–18 GHz. An RL value of −10 dB corresponded to a microwave absorption of 90% [19]. The RL value of PZW3 was lower than −10 dB in the frequency range of 17.04–17.20 GHz. Compared with the RL value of the PZW3 composite, the RL value of the PE/ZnO composite was lower. It was demonstrated that PE had no effect on the reflection loss. In Figure 6b, the three-dimensional RL image of PZW3 shows the effects of frequency and thickness on microwave absorption. The RL value of PZW3 was lower than −10 dB in the frequency range of 17.04–17.20 GHz, with a thickness range of 3–5.5 mm. Moreover, the RL value below −20 dB appeared in the frequency of 17.12 GHz, with a thickness of 3.5 mm. Thus, the deposition of ZnO NPs on the wood-fiber composite could enhance the microwave absorption properties of the composite.

**Figure 7:**
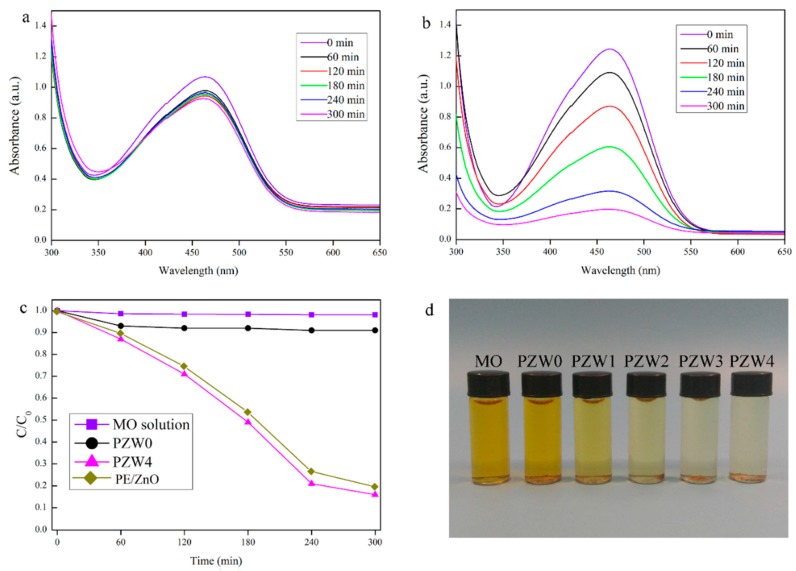
The UV spectra of MO solutions in the presence of the PZW0 powder (**a**) and the PZW4 powder (**b**) under UV irradiation. Photocatalytic activity (**c**) of MO, PZW0, PZW4, and PE/ZnO; (**d**) images of MO, PZW0, PZW1, PZW2, PZW3, and PZW4 under UV light irradiation.

The photocatalytic abilities of the as-prepared composites were estimated by the decomposition of a methyl orange (MO) solution under UV irradiation. Figure 7 shows the photocatalytic curve of the MO solution. In Figure 7a, the PZW0 composite showed a little photocatalytic ability of about 8% that increased as the exposure time increased, and that may be attributed to the physical absorption of cellulose [31]. Moreover, this result indicated that the effects of the wood fibers and of PE on the photocatalytic ability were eliminated. Figure 7b shows the photocatalytic ability of the PZW4 composite. The characteristic absorption peak of the MO solution at around 464 nm changed after UV irradiation for 60 min. The intensity of the photodegration curve of PZW4 decreased strongly as the exposure time increased. It was obderved that the addition of the ZnO NPs enhanced the degradation efficiency of the PZW4 composite. The photodegration curves of the MO solutions of MO, PZW0, PZW4, and PE/ZnO composites under UV light irradiation are displayed in Figure 7c. The photocatalytic activities of MO, PZW0, PZW4, and PE/ZnO were 3%, 8%, 84%, and 80%, respectively, after UV irradiation for 300 min. The photocatalytic activity of the PE/ZnO composite was lower than that of the PZW4 composite when the content of ZnO was the same. This indicated that ZnO plays a dominant role in regulating the photocatalytic activity. This was attributed to the fact that MO molecules were absorbed by physical adsorption on the fibers’ surface. In addition, it was observed that the photocatalytic activity derived from the ZnO NPs. The images of MO, PZW0, PZW1, PZW2, PZW3, and PZW4 after UV irradiation for 300 min are shown in Figure 7d. The experiment confirmed that the ZnO NPs enhanced the photocatalytic activity of the PE/ZnO/wood-fiber composite.

**Figure 8:**
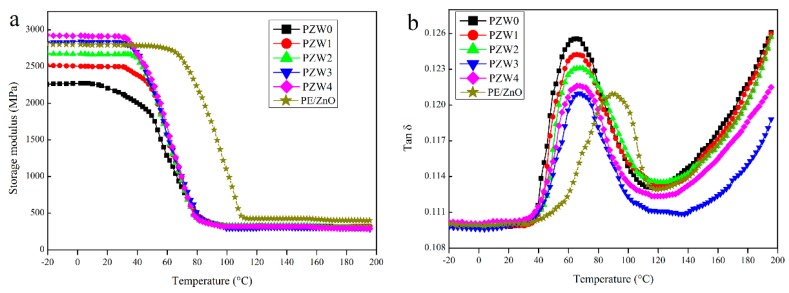
The storage modulus (**a**) and tan δ (**b**) curves of the PE/ZnO composite and the PZW composite, with different contents of ZnO NPs.

To investigate the reinforcing efficiency of the ZnO NPs on the PZW composites, the dynamic mechanical analysis (DMA) was used to measure the storage modulus and the loss factor. Figure 8 shows the dynamic mechanical properties of the PE/wood-fiber composite, the PE/ZnO composite, and the PE/ZnO/wood-fiber composite. Figure 8a displays the effect of the ZnO NPs on the storage modulus of PE/ZnO, PZW0, PZW1, PZW2, PZW3, and PZW4. Compared with PZW0, the addition of the ZnO NPs enhanced the storage modulus of the PZW composites in the temperature range from −20 to 80 °C. The reinforcing effect of the ZnO NPs could result in the increase of stiffness. The storage modulus of the PZW composites decreased below the glass transition temperature (Tg) as the temperature raised. At temperatures near Tg, a rapid decrease of the storage modulus values was observed, indicating that the PZW composites would undergo a transition stage from glassy to rubbery. The storage modulus of the PE/ZnO composite decreased as the temperature reached 80 °C. This was ascribed to the fact that the heat distortion temperature of PE was 80 °C. The PE/ZnO composite showed a high storage modulus. In the presence of the same content of ZnO, the value of the PE/ZnO composite was slightly lower than that of the PZW4 composite. Therefore, PE had an enhancement effect on the dynamic mechanical properties. Figure 8b displays the damping-factor (tan δ) curves of PE/ZnO, PZW0, PZW1, PZW2, PZW3, and PZW4. It was reported that the lower tan δ values associated with the glass transition temperature reflect the improved load-bearing properties of the system [1]. Compared with the tan δ of PZW0, the PZW composite had a low tan δ value. The peak height of tan δ decreased as the ZnO NPs increased, which indicated that the stress transfer and interface bonding between interface layers were increased [32]. The Tg value of the PZW composite increased by 4–5 °C as the ZnO NPs increased.

**Figure 9:**
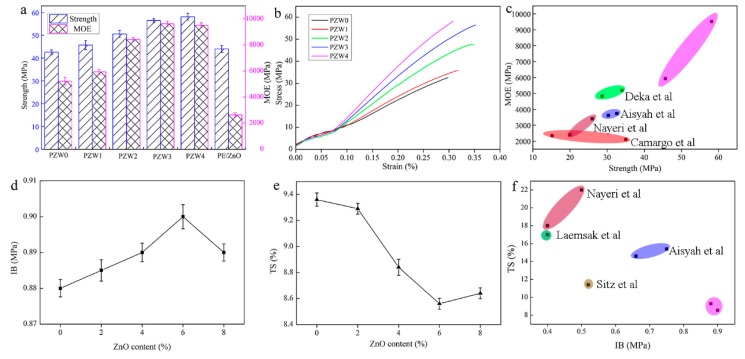
The mechanical strength histogram (**a**); stress-strain curves (**b**,**c**); IB strength (**d**) and TS curves (**e**,**f**) of PE/ZnO, PZW0, PZW1, PZW2, PZW3, and PZW4.

Figure 9 displays the flexural strength and modulus of elasticity (MOE) of the PZW composites. As shown in Figure 9a, the strength of PZW0 was 43 MPa, and the MOE was 5205 MPa. The strength and MOE values of PZW0 were far higher than those found in other studies, which was attributed to the fact that the PE molecules had a reinforcing effect on fiber materials. With the addition of the ZnO NPs, the mechanical strength increased gradually. When the content of ZnO NPs was 8%, the mechanical strength reached the maximum value of 58 MPa. Compared with that of PZW0, the mechanical strength increased by 36%. The MOE reached the maximum value of 9625 MPa when the content of ZnO was 6%, thus increasing by 85% compared with that of PZW0. The PE/ZnO composite showed a high flexural strength and a low modulus of elasticity. It was demonstrated that PE had an enhancement effect on the flexural strength of the PZW composites. Figure 9b shows the stress-strain curves of all composites with different ZnO contents. When the stress was lower than 6 MPa, the materials remained in a flexible stage (the stress was proportional to the strain). When the stress increased reaching a maximum limit, the materials started bending until they broke. Figure 9c shows that the mechanical strength of the PZW composites was higher than that of other materials. Figure 9d displays the internal bond strength of all PZW composites. The internal bonding (IB) strength of PZW0, corresponding to 0.88 MPa, reached the national standard (GB/T 11718-2009) requirements. With the addition of ZnO, the IB strength reached a maximum value of 0.90 MPa. Figure 9e exhibits the thickness swelling properties of all PZW composites after soaking in water. The distribution of the ZnO NPs on the fibers’ surface disturbed the water absorption capacity, resulting in the decrease of the thickness swelling (TS) values. The minimum TS value was 9% when the content of ZnO was 6%. These results demonstrated that the ZnO NPs had an enhancing effect on the mechanical properties of wood-fiber-polymer composites. Figure 9f displays the comparison of the mechanical properties of the PZW composites with those of other peer biomass materials, showing that the properties of the PZW composites were better than those of the other materials [32,33,34,35,36,37].

## 3. Materials and Methods

### 3.1. Materials

Wood fibers were provided by Zhejiang New Wood Material Technology Co., Ltd. (Ningbo, China). The wood fibers were derived from poplar wood, and had a length in the range of 300–1000 μm, with an average diameter of 40 μm. ZnO nanoparticles with an average diameter of 30 nm were supplied by Aladdin Industrial Corporation (Shanghai, China). Polyethylene (Grade: Ultra-high molecular weight Polyethylene, Mw = 5,000,000) was obtained from Mitsui Chemicals, Inc. (Shanghai, China). Cationic polyacrylamide (CPAM, Mw = 15,000,000) was acquired from Henan Huayang Water Treatment Materials Co., Ltd. (Zhengzhou, China).

### 3.2. Synthesis of the PE/ZnO/Wood-Fiber Composite

**Figure 10:**
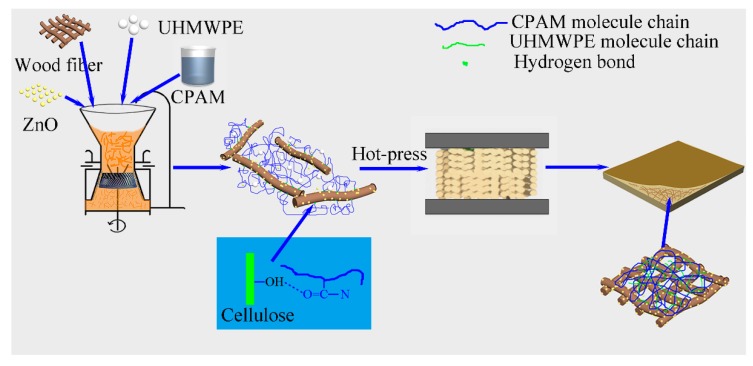
The synthesis of the PE/ZnO/wood-fiber composites.

A PE/wood-fiber mixture was soaked in 1000 mL of CPAM solution (0.01 wt %), and the mass ratio of PE to wood fiber was set as 1/10. The mixed liquid was transferred into a colloid grinder with a disc distance of 0.1 mm (Model: JM-L80, 2880 r/min, Shanghai Shen’ou General Valve Industry Co. Ltd., Shanghai, China), dispersed, and ground for 6 h. The mixed cylindrical model was obtained by vacuum filtration using a vacuum pump. The model was compressed by a hot-press at 200 °C under a pressure of 3 MPa for 25 min. In order to study the effect of ZnO content on the PZW composites, the mass ratio of ZnO to wood fiber was set as 0/50, 1/50, 2/50, 3/50, and 4/50, respectively. The as-prepared samples were designated PZW0, PZW1, PZW2, PZW3, and PZW4, respectively, according to the mass ratio of ZnO to wood fiber. The PE/ZnO composite was prepared via extrusion and injection molding, and its mass ratio of ZnO to PE was 4/50. The preparation process of the PZW composite is shown in Figure 10.

### 3.3. Characterization

The morphologies and element distributions of the PE/ZnO/wood-fiber composites were investigated by scanning electron microscopy and energy dispersive spectrometer (SEM-EDS) analysis (FEI Quanta 200, EDS/EDX Genesis, FEI Inc., Hillsborough, OR, USA). The dispersion of the ZnO nanoparticles on the fibers’ surface was investigated by transmission electron microscope (TEM, Tecnai G2 F20 S-TWIN, FEI Inc., Hillsborough, OR, USA). The X-ray diffraction (XRD) experiments were carried out in the 2θ range from 10° to 80° at a scan rate of 4°/min, using an X-ray diffractometer (Bruker D8 Advance, Bruker AXS GmbH, Karlsruhe, Germany) with Cu Kα radiation (λ = 1.5418 Å). The structure analysis based on chemical groups was performed by Fourier transform infrared spectroscopy (FTIR, Nicolet iN10 MX, Thermo Fisher Scientific, Waltham, MA, USA), with a spectrometer resolution of 4 cm^−1^, and 32 scans for signal on average. The chemical elements were determined by X-ray photoelectron spectroscopy (XPS, Thermo ESCALAB 250XI, Thermo Fisher Scientific, Waltham, MA, USA) using an ESCALab MKII X-ray photoelectron spectrometer (Thermo Fisher Scientific, Waltham, MA, USA) with Mg-Kα X-rays as the excitation source. The thermal properties of the composites were measured using a thermo-gravimetric analyzer (TGA, STA449F3, NETZSCH GABO Instruments GmbH, Ahlden, Germany) with a heating rate of 20 °C/min, up to 800 °C, under a nitrogen atmosphere.

### 3.4. Electromagnetic Test

The electromagnetic wave absorbing properties of the PE/ZnO/wood-fiber composites were measured by a vector network analyzer (Model: Keysight E5071C ENA, Agilent Technologies, Inc., Santa Clara, CA, USA) in the frequency range of 2–18 GHz. The reflection loss (RL) curves were calculated by relative complex permittivity and permeability at a given frequency and layer thickness, which can be calculated through the following equations [19]:

Z_in_ = Z_0_·(μ_γ_/ε_γ_)^1/2^·tan h·[j·(2·π·f·d/c)(μ_γ_·ε_γ_)^1/2^], (1)

RL = 20·log [(Z_in_ − Z_0_)/(Z_in_ + Z_0_)], (2)

where Z_in_ was the input impedance of the absorber, Z_0_ the impedance of free space, μ_γ_ the relative complex permeability, ε_γ_ the complex permittivity, f the frequency of microwaves, d the thickness of the absorber, and c the velocity of the light.

### 3.5. Photocatalytic Activity Test

The photocatalytic activities of the PE/ZnO/wood-fiber composites were evaluated through the decomposition of methyl orange (MO). The irradiation source was an ultraviolet 100 W lamp with a wavelength of 254 nm. Dried powders of the PZW composites were dispersed in 25 mL of MO aqueous solution with a concentration of 15 mg/L, and the PE/wood-fiber composite was used for comparison. The suspensions were stirred in the dark for 30 min to ensure the establishment of an adsorption-desorption equilibrium of the MO molecules. At set intervals (30 min), 2 mL of the suspension was extracted and then centrifuged to separate the catalysts from the supernatant. The degradation efficiency of MO was measured by UV-vis spectrophotometer (Pgeneral TU-1901 UV-Vis spectrophotometer, (Beijing Puxi General Instrument Co., Ltd., Beijing, China)); the maximum absorption wavelength of MO was 464 nm.

### 3.6. Dynamic Mechanical Analysis

The dynamic mechanical properties of the PE/ZnO/wood-fiber composites were determined using a dynamic mechanical thermal analyzer (DMTA, TA Q800, TA Instruments, New Castle, DE, USA) in a three-point bending system; the samples dimensions were 50 × 10 × 4 mm. The samples were measured in an air atmosphere at a fixed frequency mode of 5.0 Hz, and oscillation amplitude of 15 μm. The samples were evaluated in the temperature range of −20 to –200 °C with a heating rate of 2°/min.

### 3.7. Mechanical Studies

The flexural properties of the PE/ZnO/wood-fiber composites were measured with the universal testing machine (Model: MWD-100, Jinan Asahi Instrument Equipment Co., Ltd, Jinan, China) using the three-point bending test at a loading rate of 5 mm/min. The experiment was carried out according to the Chinese Standard GB/T 11718-2009. The samples’ dimensions were 100 × 20 × 5 mm, and the number of test pieces was 15. Fifteen test samples of 50 × 50 × 5 mm were prepared for the internal bonding (IB) strength test, which was performed using the universal testing machine (Model: MWD-100, Jinan Asahi Instrument Equipment Co., Ltd, Jinan, China) at a loading rate of 5 mm/min. Fifteen samples of the same size were examined in the thickness swelling (TS) test, after 24 h of immersion in water at 20 °C.

## 4. Conclusions

ZnO NPs were deposited on PZW composites through the precipitation of CPAM during a grinding process, and the final PZW composites were obtained via hot-press. The PZW composites showed EM absorption properties and superior mechanical properties, and could degrade MO under UV irradiation. The minimum-reflection-loss value of the PZW composites was −21 dB, with a thickness of 3.5 mm in the frequency of 17.17 GHz. The degradation efficiency of the PZW composites was about 84% under UV light irradiation. The synergistic effects of the ZnO NPs and PE enhanced the mechanical strength of the composites. The value of the storage modulus of the PZW composites increased as the content of ZnO increased. It was found that the effectiveness of interfacial stress transfer was increased. The peak height of tan δ was lower in the PZW composites, which indicated lower damping and good adhesion. The flexural strength of PZW4 was 58 MPa, and the MOE of PZW3 was 9625 MPa; the IB and TS values of PZW3 were 0.88 MPa and 9%, respectively.

## Figures and Tables

**Table 1:** Element content and C/O ratios of the PE/ZnO/wood-fiber composite.

Sample	C (at %)	O (at %)	N (at %)	Zn (at %)	C/O Ratio
PZW0	61.35	34.54	4.11	-	1.78
PZW1	57.95	35.46	4.10	2.49	1.63
PZW2	53.16	38.74	4.09	4.01	1.37
PZW3	49.67	39.95	4.03	6.35	1.24
PZW4	47.56	39.67	3.96	8.81	1.19
